# Discharged. Not Where to Lay His Head

**Published:** 1886-10-09

**Authors:** George Fleming


### DISCHARGED.

### "NOT WHERE TO LAY HIS HEAD."

By George Fleming.

II.

It was one of those four or five warm afternoons at the end of last October. The veiled delicate gold of the autumn sunshine, the transparent delicate blue and white of the autumn sky, cast a grace not its own about the straight line and solemn uncompromising respectability of Exhibition Road. There were very few people in the street. The great brick houses were all shut and barred; but Lemon did not question for one moment the ultimate success of his mission. Miss Thornhill had promised to find him work, and he was going at once to look after it; none of your loitering over a job for him! but, meanwhile, the cup of hot coffee that he stopped to drink at the station had put new vigour into his shaking body. "None of your beer for *me*. No, thank you, Miss. I'm a temperance man now; that's about my ticket!" he had said to the young woman who served him at the bar: and when she smiled, looking at his handsome face, he had laughed too. A feeling of vague, but warm but illimitable friendliness towards the whole human race seemed to pervade the air he breathed, filling and transfiguring his inner world of being as the gracious sunlight filled the sky. At the corner, by the Museum, two policemen were standing talking to one another. "I never saw such weather. I never did," one of them was saying, looking up at the sky as Lemon passed; and he smiled to himself with pleasure over the words, as if this glory of ample light were in some way his doing, his gift of goodwill to all his fellow men.

He walked very slowly. The gentleman in the train had given him some tobacco; he stood for some time, smoking, before the closed entrance to the Healtheries. They had read all about it in the ward, and he could not help wishing he had got out a week or two sooner in time to have a look at it, so as to be able to tell his mate. While he was still leaning there against one of the pillars, a little black-and-white dog came trotting down the middle of the empty street, scampering gaily along on three legs with one small paw in reserve. Lemon whistled to him. "Hi, Spotty! come here, you Spot!" he said. He stopped to play with him for several minutes, and when the friendly little mongrel showed signs of growing tired of leaping and barking, Lemon could not resist the temptation of whistling him back. "Like as not, he's lost. They'd only run him in at Battersea," he said to himself apologetically.

It was nearly six o'clock before he reached the house, the address of which Miss Thornhill had given him. As he crossed the street he noticed that all the blinds were drawn down. For the first time, he began to feel a little anxious. He rang twice; three times; at the front door bells, at that marked "Visitors" and "Servants" alternately, before anything stirred in the house. Finally there was a sound of hesitating steps in the hall. Lemon wiped the perspiration from his face, and took off his old black hat. The door opened a few inches, with the chain up, and a fresh, girlish voice demanded, "Who is there?"

"Beg pardon, but does Miss Thornhill live here, if you please, miss?"

There was a pause of a minute or two, with the door still held ajar, and he repeated his question.

"Miss Thornhill gave me this address, and told me to call. My name's Lemon. I'm just out of the hospital. I'd like to see her if it's convenient."

"What—what do you want?" said the voice, and at the same instant the chain was slipped and the door opened wide. A young girl of sixteen or seventeen, wearing the neat print frock and white cap of an under-servant, a girl with rough curly hair, and a pretty face, stood before him. She had a frightened look; glancing nervously up and down the street, and keeping her hand upon the door, evidently ready to slam it in case of emergency.

"Miss Thornhill ain't at home. She's gone away," she said hesitatingly, looking first at Lemon and then at his dog.

"Gone away? Why—why, she told me to come here. She gave me this address. Look a here! there 'tis, as she wrote it!" Lemon cried out huskily, in a sudden fit of terror.

He had left the Infirmary a few hours before with more than a shilling in his pocket (it was money Miss Thornhill had given him at different times to buy stamps, and he had never written the letters); but there had been the fare up from Rotherhithe, and then that coffee at the station.

He thrust his hand into his pocket and felt for the loose pence. "Gone away!" he repeated, mechanically.

In a moment all the strength seemed to be taken out of him. After seven months of lying in bed, his legs shook under him with the effort of walking. He had been standing there all this time holding his hat in his hand, but now he thrust it on his head again and leaned up against the door-post, staring at the little servant with blank, incredulous eyes. He was weak, he was tired, he was hungry; he had no work; he did not know where to look for a night's lodging; he had about sevenpence halfpenny in his pocket, and Miss Thornhill was out of town.

"Well, that about does for me," he said, after a minute or two of this silence.

The little black-and-white dog leaped up against him to lick one of his hanging, bloodless hands, and he stared at the small creature as if he had never seen it before.

"I came here—I'm just out o' hospital. And there warn't no place to go to as I know of," he said, heavily, after another pause.

And then suddenly, feverishly, without a reason for it, he began to tell her the whole of his story. The girl listened in silence, looking up into his face every now and then, the tears gathering slowly in her soft, dove-like eyes. She stood listening on the door-step, a humble little Desdemona, while the low afternoon sun touched the bright knot of ribbon at her throat and made a golden halo of her rough curly hair.

---

III.

He told her all the story of his illness. But he did not stop at that: he went back further. He told her what good wages he had been in, and all about the Rothschild job, and how he had married on the strength of it—ay, and kept his wife like a lady too,—until—well, first she fell sick, she was always delicate, and then the two children never properly got over the scarlet fever. When their mother died there was no one to look after the house; and work got slack. "It's an awfully good trade for paying, but it's chancey; chancey, that's what it is—up and down hill work. And then," he said, "things went on growing worse and worser. I got this cough. And the children—one of them, the little girl that was, had hair just like yours, miss. There was a lady—I was living down at the East End then, Shadwell way; perhaps you know it? Ah, well, I'm a West End man myself. But, as I was a sayin', the clergyman's wife, she got her into a Home down there, but it warn't no good. They was both of 'em as delicate as their mother; both of 'em."

The girl drew a long breath.

"Ah," she said, "you've had a deal of trouble!" Her eyes dropped. "You're not so old to look at either. I'd never have thought you had been married."

"I'm goin' on for thirty, though. That's about my ticket," Lemon said with a vague laugh.

He was silent for a moment. Then he went on.

"I've had some hard times of it. That's true too. But Lord bless you, there's trouble and trouble. There's what comes; and there's what it seems as if a man went out of his way to fetch into his life. I've had time to think a heap about them things lately,—while I was gettin' well. I've had a fall, I have; but I ain't done for yet. All I want is just some time, a week maybe, to look about me and get on my feet again. And I'm a temperance man now, miss. I've got the pledge all signed, there, in my pocket 'Twas to please Miss Thornhill too as I signed it: she began at me about it one day—" And then, without any warning, without well knowing himself why he spoke, he poured out into her willing ears all the history of his downfall. He told her about his drinking; and the queer company he had kept; until, one by one, he had pawned each bit of the furniture his wife had bought with her savings in the first months of their marriage. "The doctor found me,—not our Dr. White I was a tellin' you of; *he* is House Surgeon; but one of the outside doctors,—he found me starving in a cellar in Rotherhithe, an' that's the undoubted fact, an' as such ain't to be contradicted," he said, bringing his hand down hard against his knee. The little spotted dog leapt to his feet with a shrill bark of joy, and the girl looked up at him with her pretty, tender eyes.

In all the seven months he had lain ill in that workhouse, through all those long, purposeless, demoralising afternoons, when idle, slipshod men shuffled about the ward from bed to bed in endless barren talk, he had never let out one-half the truth about himself which he poured out now, unsolicited, into the sympathetic ears of this little maid. She listened in perfect silence. It was only when he had come back to the subject of Miss Thornhill's absence that she seemed to awaken, as it were, from her attentive trance.

Then indeed she gave him brief advice and good. Miss Thornhill was in the country with her father and all the upper servants. "I don't know her address, but

I can get it from my aunt. She's out now. She is the cook here, and I help her in the kitchen. We're left on board wages to look after the house. You write to Miss Thornhill and I'll see that she gets it. Maybe it isn't so far off in the country. And she's an awfully kind lady; she's sure to answer back immediate."

She turned her head aside and began polishing the door-knob with the hem of her apron.

"Is that dog yours?" she asked abruptly.

"No. But he's followed me, like. He's a nice little chap, too," Lemon said, pushing the little animal gently about with his foot.

"Oh. I thought maybe he was yours; that he'd been with you in the hospital. If I were you," she said, looking up suddenly, "I'd go to one of them coffee taverns to-night. There's one where they let rooms, too, in Knightsbridge. It ain't far." She blushed all over her face and neck. "And then you can bring your letter over here to-morrow. Aunt's mostly out at this hour. Aunt is country bred. She don't like me talking to strangers."

"Very well," Lemon answered, "I will. And I'm ever so much obliged to you. I won't forget it."

She looked so pretty standing there all flushed and glowing in the low sunlight, that involuntarily he began to smile. "I shan't forget it," he repeated vaguely, looking up and down the street; "when I get on my feet again like a man— I'd like to know what your name is. I'd like to know what name to think of you by."

"My name? I ain't got anything to do with it. Mary Parker," she said, beginning to laugh too, in her turn.

When she had shut the door, oh, so softly and gently, Lemon went down the steps. The small black-and-white dog marched gaily after him, cocking his shabby little tail well up in the air, and after a minute or two the young fellow broke out into a loud, melodious whistle. It was only some tune from a music-hall, but he whistled it like a bird.

At this hour, down there at the workhouse, the bell would be ringing for the men's tea. As he strolled along through the mellow light in that clean, finely tempered air, he fell to thinking of the face of the man who had slept for so long in the next bed to his own. He could see him now, poring through his spectacles into his brimming tea-cup. That neighbour of his was an old hand in the House; as Lemon grew stronger the two men had had many and many a yarn together at this hour. He thought of it now; but it seemed as if a hundred years had gone by since this morning—since he had left all that behind him.

(*To be continued.*)

